# “The sky on our shoulders”: a qualitative study of family caregivers’ psychosocial experiences and unmet palliative care needs in advanced lung cancer

**DOI:** 10.1186/s12904-026-01991-8

**Published:** 2026-01-20

**Authors:** Chuntong Shen, Fengxia Liu, Xuejing Mu, Ting Zhang, Liman Wang, Jian Shi

**Affiliations:** 1 Department of Oncology, The Fourth Hospital of Hebei Medical University, Health Road, Shijiazhuang City, Hebei Province 050000 China; 2 Nursing Department, The Fourth Hospital of Hebei Medical University, ShiJiaZhuang, 050000 China

**Keywords:** Advanced lung cancer, Family caregivers, Chemotherapy, Psychosocial experience, Supportive care needs, Qualitative research, Palliative care

## Abstract

**Background:**

Family caregivers serve as the cornerstone of care for patients with advanced lung cancer during chemotherapy, carrying a profound psychosocial burden. Little is known about their lived experiences and unmet needs within the distinctive Chinese cultural context. Elucidating these needs is crucial to inform the development of culturally sensitive, family-centered palliative care models in China.

**Methods:**

We conducted a qualitative phenomenological study. Fourteen family caregivers were purposively recruited from a tertiary hospital in China. In-depth, semi-structured interviews were conducted face-to-face between July and August 2024, continuing until thematic saturation was reached. Interviews were audio-recorded, transcribed verbatim, and analyzed thematically following Colaizzi’s seven-step method.

**Results:**

Four overarching themes were identified: (1) Profound Information Needs Amidst Uncertainty; (2) The Cascading Burden of Distress: Emotional Turmoil and Unmet Support Needs; (3) Adaptive and Burdened Caregiving Behaviors; and (4) Seeking Anchors: The Critical Role of Support Systems.

**Conclusion:**

This study reveals the “sky on our shoulders” of Chinese caregivers—a burden defined by multifaceted unmet needs that highlight critical gaps in palliative care support. To address these gaps, palliative care services must integrate systematic assessment and tailored interventions for caregivers’ informational deficits, profound emotional turmoil, and need for coping skills and resources. Healthcare professionals are essential in validating this burden and providing culturally congruent, sustained support throughout the care trajectory.

**Supplementary Information:**

The online version contains supplementary material available at 10.1186/s12904-026-01991-8.

## Introduction

Statistics from the National Cancer Center indicate that in 2022, China had approximately 1.06 million new cases of lung cancer and 733,300 lung cancer-related deaths, accounting for 22% and 28.5% of all malignant tumor incidence and mortality, respectively. Lung cancer remains one of the major malignant tumors threatening human health [1]. Recent advancements in treatment methods and technologies have resulted in an increase in the 5-year survival rate for lung cancer in China, rising from 16.1% to 19.7% [2]. However, 75% of patients are diagnosed with advanced lung cancer [3]. The prognosis for patients with advanced lung cancer is poor, and chemotherapy is the most common treatment modality. Although chemotherapy effectively inhibits tumor growth, it also induces toxic side effects that impair patients’ quality of life and increase the burden on caregivers [4].

In recognition of this burden, the integration of early palliative care—aimed at mitigating suffering and improving quality of life for both patients and families—has become a cornerstone of advanced cancer management internationally. However, in China, family-centered palliative care remains underdeveloped, and family members continue to serve as the cornerstone of long-term care, shouldering immense responsibilities encompassing medical decision-making, daily care, emotional sustenance, and financial support [5, 6]. This critical role often comes at a significant personal cost, placing caregivers at high risk for substantial psychosocial burden and unmet supportive care needs [7]. Alarmingly, as many as 50% of caregivers for lung cancer patients experience clinically significant symptoms of anxiety or depression [8].

The distress experienced by caregivers is not merely an individual concern; it is a clinically relevant issue. caregiver distress can directly compromise their own health and critically impact the quality of care delivered, ultimately influencing patient outcomes such as treatment adherence and survival [9, 10]. Therefore, addressing the multidimensional needs of caregivers is not only an ethical imperative but also essential for optimizing patient-centered and family-focused palliative care.

While the challenges faced by cancer caregivers are globally recognized, their manifestation is profoundly shaped by cultural context. In China, deeply ingrained Confucian values, such as filial piety, dictate strong familial obligations [11]. This cultural script influences everything from caregiving motivation and communication patterns (notably the dilemma of truth-telling) to the suppression of one’s own emotions to maintain family harmony. Although the salience of this cultural context is acknowledged [12], studies that explicitly employ a theoretical framework to analyze how these specific norms—filial piety, family harmony, and truth-telling—dynamically shape the stress-coping processes of caregivers during advanced cancer chemotherapy remain scarce. Consequently, a nuanced understanding of how these cultural forces mediate caregivers’ appraisal of stressors, their emotional responses, and adaptive behaviors is underdeveloped.

Guided by the Stress and Coping Framework [13], which elucidates the interplay between cognitive appraisals, emotional responses, coping resources, and behavioral adaptations, this qualitative study has a dual aim: First, to provide a nuanced understanding of the lived psychosocial experiences and specific unmet needs of Chinese family caregivers. Second, and as its primary theoretical contribution, to critically explicate how the aforementioned Chinese cultural norms fundamentally shape and are reflected in each stage of the stress-coping process across the chemotherapy trajectory. This culturally-informed analysis seeks to move beyond description, aiming to provide a contextualized explanation for the observed patterns of distress and resilience that is vital for designing effective and congruent palliative care interventions in China.

## Methods

### Study design and ethical considerations

This qualitative phenomenological study was conducted in the oncology ward of a tertiary hospital in Shijiazhuang, Hebei Province, China. It was approved by the Ethics Committee of the Fourth Hospital of Hebei Medical University in accordance with the declaration of Ministry of Health’s Measures for Ethical Review of Biomedical Research Involving Human Beings (2016), the WMA Declaration of Helsinki (2013) (Registration number:2024KS144), and all participants provided informed consent and voluntarily participated in the study.

### Research team and reflexivity

The research team was purposively composed to incorporate multidisciplinary perspectives, including oncology, nursing, nursing administration, and psychosocial care. All members received training in qualitative research methods. The primary researcher (CS), an oncology nurse, conducted the interviews and preliminary analysis; her clinical background facilitated rapport and in-depth engagement with participants. To minimize potential bias and ensure analytical rigor, two researchers (CS and XM) performed independent coding and thematic development. The team held regular meetings to discuss and resolve coding discrepancies, with a senior researcher (JS) consulted for arbitration when necessary. Throughout the process, the primary researcher maintained a reflective journal to consciously bracket clinical presuppositions and enhance reflexivity.

### Participant recruitment and sampling

From July to August 2024, fourteen family caregivers were purposively recruited. The inclusion criteria were: (1) caregivers of patients diagnosed with advanced lung cancer undergoing chemotherapy confirmed by pathology; (2) aged ≥ 18 years, including parents, spouses, children, and siblings; (3) caregivers in good physical health with normal mental and behavioral capabilities; (4) voluntary participation in the study without language communication barriers. The exclusion criteria were: (1) history or existing mental disorders or cognitive impairments; (2) caregivers receiving compensation. The sample size was determined through data saturation. Recruitment continued until thematic saturation was achieved, which was defined as the point when no new themes or sub-themes emerged from the last three consecutive interviews, and all existing themes were fully developed and nuanced. Saturation was reached after fourteen interviews.

### Development of semi-structured interview outline

A semi-structured interview guide (supplementary Table 1) was developed for this study based on a literature review, internal research team discussions, and pilot interviews with two caregivers. The guide was refined for clarity and relevance before final use.

The main interview questions explored the following areas:

1. Since the patient’s diagnosis, what information about the illness or caregiving have you found yourself searching for? What questions remain unanswered for you?
2. How has the role of being a caregiver affected your daily life, your work, and your relationships with other family members?
3. What negative emotions have you experienced during the caregiving process? How did you adjust?
4. What difficulties have you encountered while providing care?
5. What personal strategies or external resources have you found helpful in overcoming these challenges?
6. In the caregiving process, which areas do you anticipate healthcare professionals to provide support and assistance?

### Data collection

Data collection was conducted through face-to-face, semi-structured interviews carried out by the researcher with participants in a quiet conference room within the Department of Oncology. Interviews typically lasted 20 to 45 min. During the interviews, the interviewer followed the interview guide, employing techniques such as active listening and appropriate probing to encourage caregivers to express their thoughts in depth, without suggesting or leading their responses. Additionally, significant expressions and body language displayed by the caregivers during the interviews were documented.

### Data analysis

Interview recordings were transcribed verbatim within 24 h. All transcripts were anonymized with sequential codes (P1–P14) and analyzed following Colaizzi’s seven-step phenomenological method [14]. Two researchers (CS and XM) independently conducted the analysis, which involved: (1) repeated reading for immersion; (2) extraction of significant statements; (3) formulation of meanings; (4) clustering meanings into themes and sub-themes; (5) development of a detailed narrative description; (6) distillation of a core structural essence; and (7) validation through participant feedback, which led to confirmatory revisions. To ensure analytical rigor and trustworthiness, multiple strategies were employed. First, participant validation (member checking) was performed by sharing preliminary thematic summaries with four participants (P1, P4, P7, P8), who were selected to represent a range of caregiver relationships (spouse, child, daughter-in-law) and educational backgrounds. Their feedback was used to confirm interpretive accuracy and refine the thematic structure. Second, analyst triangulation was achieved through independent coding by two researchers (CS and XM), with regular team discussions to resolve discrepancies and consultation with a third senior researcher (JS) for arbitration when needed. Third, reflexivity was maintained through the primary researcher’s reflective journal to bracket clinical preconceptions. Fourth, non-verbal cues and emotional tones documented during interviews were integrated into the analysis. Finally, a comprehensive audit trail was maintained, detailing all analytical decisions from initial coding to final theme development, thereby ensuring the transparency and confirmability of the research process.

## Results

Data saturation was reached after fourteen interviews.Their ages ranged from 24 to 71 years (mean,46.1[SD,14.1]years). The average age of the patients was 57.1 ± 6.8 years. The specific information is shown in Table 1. Four themes were formed: (1) profound information needs amidst uncertainty, (2) the cascading burden of distress: emotional turmoil and unmet support needs, (3) adaptive and burdened caregiving behaviors, and (4) seeking anchors: the critical role of support systems .Themes and sub-themes are shown in Table 2.

**Table 1:** Characteristics of caregivers and patients

NO	Family Caregiver					Patient		
	Gender	Age (years)	Educational level	Occupation	Relationship with patients	Age (years)	Disease staging	Chemotherapy cycle
P1	Female	38	Junior high school	Freelance work	Daughter-in-law	61	Ⅳ	1
P2	Male	65	Primary school	Farmer	Spouse	61	Ⅳ	3
P3	Male	66	High school	Retired people	Father	42	Ⅳ	3
P4	Male	36	Regular college course	Staff	Son	64	Ⅳ	8
P5	Male	40	Junior college	Freelance work	Son	61	Ⅳ	2
P6	Male	48	Primary school	Worker	Spouse	46	Ⅳ	1
P7	Female	58	Primary school	Farmer	Spouse	60	ⅢB	1
P8	Female	35	Junior high school	Freelance work	Daughter	54	Ⅳ	4
P9	Female	57	Junior high school	Farmer	Spouse	55	ⅢC	3
P10	Female	24	High school	Unemployed	Daughter	53	Ⅳ	2
P11	Male	32	High school	Staff	Son	57	Ⅳ	2
P12	Female	38	High school	Staff	Daughter	58	Ⅳ	1
P13	Female	37	Regular college course	Nurse	Daughter	58	Ⅳ	3
P14	Female	71	Junior high school	Farmer	Spouse	70	Ⅳ	6

**Table 2:** Themes and Subthemes

Themes	Subthemes
Profound Information Needs Amidst Uncertainty	Varied Levels of Baseline Knowledge Shaping Cognitive Appraisals
	Strong Desire for Clear and Accessible Information
	Chemotherapy-Related Fears and Misconceptions
The Cascading Burden of Distress: Emotional Turmoil and Unmet Support Needs	The Emotional Onslaught: from Shattering Shock to Burdensome Adaptation
	The Culturally-Embedded Dilemma: Truth-Telling as a Central Stressor
	The Dual Torment: Illness Anxiety and Financial Toxicity
	Powerlessness and a Foreshortened Future
Adaptive and Burdened Caregiving Behaviors	Fulfilling Filial or Familial Obligations
	Navigating Difficult Communication
	Adapting to Role Shifts and Increased Responsibilities
	Emotional Restraint and Self-Silencing
	Engaging in Self-Care and Coping Efforts
Seeking Anchors: The Critical Role of Support Systems	Strengthened Family Bonds and Cohesion
	Affirming Patient Value and Dignity
	Seeking Professional Guidance and Support

### Theme 1: profound information needs amidst uncertainty

Caregivers grappled with significant uncertainty regarding the disease and treatment, highlighting an acute need for clear, accessible information to reduce fear and guide care. This lack of knowledge and understanding amplified their anxiety and burden.

### Varied levels of baseline knowledge shaping cognitive appraisals

Substantial heterogeneity was observed in caregivers’ pre-existing understanding of lung cancer and chemotherapy. Caregivers with prior experience exhibited a greater depth of knowledge regarding these topics.A nuanced understanding of treatment toxicities and management strategies.

*Having lost my uncle to small cell lung cancer, I recognized the rapid progression. (P1).*

*My uncle experienced severe nausea and vomiting during his chemotherapy—we purchased the antiemetic medication from here. (P4)*.

However, structural limitations—including advanced age, low educational attainment, and digital illiteracy—hindered partial caregivers from proactively acquiring medical knowledge, thereby reinforcing their dependency on healthcare providers.This passive reliance underscored their vulnerability and unmet need for tailored, comprehensible health information.

*Most of the knowledge I don’t fully grasp*,* I can’t read medical terms…just follow the doctor’s guidance. (P2)*.

*I haven’t searched for information online; I come here solely to listen to the doctor*,* When doctors explain too much*,* I get lost. (P14)*.

*I don’t understand the examination reports… it’s all confusing. (P6).*

### Strong desire for clear and accessible information

Caregivers consistently highlighted their existential need for clarity when facing uncertainties in treatment. This need manifested as an urgent pursuit of actionable information to establish predictability—especially concerning chemotherapy cycles, symptom trajectories, and home-care protocols. They expect healthcare professionals to communicate details about the illness, subsequent treatment plans, and management of adverse reactions in a clear, concise, and comprehensible manner. Furthermore, some caregivers actively seek relevant information online to deepen their understanding.These expressions directly articulated a critical need for proactive, patient-centered communication from healthcare providers.

*I want to know how many cycles of chemotherapy are generally required and what reactions might occur afterward. (P7)*.

*I would appreciate it if the doctor could explain it to me*,* after which I will look up some related information online. (P11)*.

*I hope you can elucidate the adverse reactions following medication so that we can understand what to monitor at home. (P14)*.

### Chemotherapy-related fears and misconceptions

Caregivers’ resistance to chemotherapy often arises from a preemptive terror rooted in the anticipation of catastrophic symptoms. Limited knowledge exacerbates the perception of imagined threats and hidden toxicity. This fear-behavior pathway directly hinders timely intervention.These fears, rooted in information gaps, demonstrate a clear need for early education and myth-busting interventions to facilitate timely and informed decision-making and reduce anticipatory distress.

*We refused chemotherapy at first, fearing unbearable vomiting would destroy him. (P1).*

*At first, we were particularly opposed to chemotherapy. (P4).*

*After chemotherapy*,* I constantly worry about adverse reactions because my understanding of this treatment is limited. (P12)*.

### Theme 2: the cascading burden of distress: emotional turmoil and unmet support needs

Family caregivers traversed a turbulent emotional landscape, where the initial shock of diagnosis evolved into a persistent state of compounded distress. This burden was uniquely intensified by deeply ingrained cultural imperatives and profound socioeconomic pressures, revealing a critical need for multifaceted support systems.

### The emotional onslaught: from shattering shock to burdensome adaptation

The diagnosis of advanced lung cancer delivered a catastrophic blow to family caregivers, precipitating an immediate emotional crisis. Their initial appraisal was one of profound shock and disbelief, which utterly shattered their sense of normalcy.

*When the parents of a former colleague were diagnosed with this disease*,* it may have seemed like just another illness. However*,* when such a situation occurs personally*,* it feels as if the sky has fallen. (P11)*

*Initially, I felt disbelief, particularly struggling to accept that he truly had this disease. (P13)*

This acute shock rapidly gave way to intense waves of sorrow for the patient and, for many, a persistent undercurrent of guilt. Caregivers anguished over their perceived failure to have prevented the illness or to have detected it at an earlier, more treatable stage.

*When the diagnosis was first made*,* seeing him in a hospital gown made me feel extremely distressed (crying…). (P8)*

*He didn’t have a follow-up check when he returned during the Spring Festival; if we had done it earlier*,* perhaps it could have been detected in time (sighing). (P3)*

As the initial turmoil persisted alongside intensifying caregiving demands, participants described entering a protracted and arduous process of psychological adaptation—a forced reconciliation with a harsh reality.

*I had to reconcile with what was challenging to accept. (P9)*

*I am slowly reconciling with this situation and collaborating with the doctors in the treatment… (P11)*.

### The culturally-embedded dilemma: truth-telling as a central stressor

The decision of whether to disclose the cancer diagnosis to the patient constituted a profound cultural dilemma, placing caregivers in a morally solitary position and intensifying their distress.

Most caregivers chose disclosure, believing it necessary for treatment cooperation and viewing concealment as impractical. This decision, however, often triggered intra-family conflict.

*He’s educated—a university graduate. At that time*,* it was impossible to hide it… But my mother blamed us for telling him*,* fearing he couldn’t cope. (P13)*

Conversely, others concealed the diagnosis to protect the patient from despair and to guard the family against severe social stigma.

*He doesn’t know his condition; I haven’t told him. (P12)*

*Given his condition*,* we avoid letting villagers know. People see this illness as a death sentence—they’d think our family is doomed. Besides*,* our children still need to attend school. Disclosing helps no one. (P3)*

Thus, regardless of the path chosen, caregivers bore the sole, weighty responsibility for this decision, transforming truth-telling into a central and culturally-specific stressor.

### The dual torment: illness anxiety and financial toxicity

Caregivers were caught in a dual storm of distress. The first arose directly from the illness: a relentless anxiety about rapid progression, debilitating side effects, and an uncertain prognosis.

*Just days ago he had a strong appetite*,* but now he can’t eat at all. (P1)*

*After the relapse*,* both lungs show metastases. Chemotherapy’s success now depends entirely on his body’s response. (P3)*

The second, and often more crushing storm, was financial. The overwhelming responsibility for medical decisions, daily care, and family needs converged with severe financial toxicity, creating an inescapable objective burden that threatened the very foundation of care.

*The financial burden is overwhelming; the medical expenses are divided between the two brothers. (P1)*

*As a father*,* I am currently covering all medical costs. Once my funds are exhausted*,* I cannot incur further debt… (P3)*.

*I cannot afford the treatment; conservative management is our only option. I haven’t earned much over the past two years*,* and I also have to cover my child’s expenses. (P6)*

This financial strain was inextricably linked to their core caregiving responsibilities, often forcing impossible choices between providing optimal care and maintaining family subsistence.

### Powerlessness and a foreshortened future

The relentless pressures of caregiving frequently eroded caregivers’ sense of control and efficacy. Struggling to balance competing obligations and facing situations beyond their influence, they experienced profound helplessness and perceived their efforts as futile.

*My wife’s condition forces me to take leave—I can’t focus on work at all! (P6)*

*Between accompanying Dad for treatment and the children’s school runs*,* I’m constantly torn in multiple directions. (P8)*

*After her leukopenia diagnosis, she refused further treatment—ignoring all my advice. (P2)*

This erosion of agency ultimately collapsed their temporal horizon. The future became too uncertain or fraught to plan for, narrowing their focus to day-to-day survival and immediate crises.

*We’re taking things one step at a time! Last hospitalization drained our savings—I can’t afford ongoing treatment. (P6)*

*Should he pass*,* I’ll still farm my land*,* though I’ll need hired help for harvest. (P7)*

### Themes 3: adaptive and burdened caregiving behaviors

Fulfilling demanding caregiving roles involved both adaptive efforts and significant burdens. Caregivers navigated complex communication, undertook major role shifts, suppressed their own emotions, and attempted self-care, all while striving to meet deeply held cultural obligations of filial piety. These experiences reveal needs for communication skills training, role transition support, and strategies to mitigate emotional suppression.

### Fulfilling filial or familial obligations

Upon discovering their parents’ illness, the children, acting as caregivers, will diligently demonstrate filial piety by aiding their family in overcoming obstacles, offering support and companionship to their parents, and fulfilling their parents’ desires. This strong sense of filial duty, while a source of motivation, also represents a significant cultural expectation and potential source of stress, necessitating culturally sensitive support that acknowledges this value.

*My mother underwent her first chemotherapy session*,* and my sister expressed her desire to accompany her. My sister possesses a carefree personality and can uplift her spirits. (P4)*.

*At his age*,* after dedicating himself to raising us*,* he deserves to receive the necessary treatments; ultimately*,* we must fulfill our filial responsibilities. (P11)*.

*There was a period when he particularly enjoyed going out and traveling. Therefore*,* my brother frequently took him on trips. (P13)*.

### Navigating difficult communication

Caregivers and patients encountered significant communication challenges regarding disease treatment, condition discussions, and daily activities. Some caregivers accommodated patients’ increased reticence or preferences, others actively engaged to alleviate psychological burdens, and many dyads intentionally avoided discussing cancer-related topics. These communication patterns highlight the need for skills training to facilitate open and supportive dialogue within the family unit.

*She has become less communicative, and I find myself having to comply with her wishes. (P2).*

*I talk to my father to comfort him. I worry that he might feel overwhelmed… concerned about burdening us. (P12)*.

*He typically avoids going out, especially initially; we rarely discuss his illness at home. (P14).*

### Adapting to role shifts and increased responsibilities

Caregivers transitioned from previous roles to assuming new responsibilities encompassing the patient’s daily life management and psychological well-being. This shift involved taking on practical tasks and altering relational dynamics. Managing this substantial increase in responsibilities underscores the need for practical support and resources to ease the caregiving load.

*She used to handle all the housework*,* but now I have taken over… she can’t tolerate the odor of cooking oil. (P2)*.

*In the past*,* we used to frequently argue… however*,* I now find myself reluctant to engage in conflicts… how many years do we have left? (P14)*

### Emotional restraint and self-silencing

Caregivers frequently suppressed their own emotions and neglected their needs to shield the patient from distress. They feared their emotional expression could negatively impact the patient’s well-being. This chronic emotional suppression poses a risk to caregiver well-being, emphasizing the need for safe outlets and validation of their own emotional needs.

*Caring for her is particularly exhausting*,* and there appears to be no effective method to manage it. (P2)*.

*If you’re feeling unwell*,* the elderly person will undoubtedly perceive this…; as for myself*,* I merely suppress my emotions. (P4)*.

### Engaging in self-care and coping efforts

Caregivers commonly experienced fatigue, poor sleep, and significant psychological stress. They employed various self-regulation strategies, including physical activity, social interaction, distraction, emotional release, and medication. While caregivers employed various coping strategies, these efforts were often sporadic, indicating a need for accessible, structured self-care support and stress management resources.

*Occasionally*,* I participate in physical exercise; sometimes I play games to relieve stress*,* or I converse with family members in the ward to release tension. (P11)*.

*Shopping can enhance my mood or I might choose to confide in my husband and share the troubles weighing on my heart. (P13)*

*I release my emotions through crying; ever since my father-in-law was diagnosed with this illness*,* I have wept numerous times.* *(P1)*.

*I took traditional Chinese medicine for a period to alleviate my insomnia. (P12).*

### Theme 4: seeking anchors: the critical role of support systems

Caregivers actively sought and relied on support systems to mitigate their burden. Strengthened family bonds provided crucial emotional and practical backing, affirming the patient’s dignity offered shared meaning, and turning to healthcare professionals represented a vital unmet need for reliable information and guidance.

### Strengthened family bonds and cohesion

Following a patient’s diagnosis, caregivers frequently experience helplessness and initiate discussions with kin or social networks to develop coping strategies. This collaborative support enables caregivers to prioritize patient care, reinforcing family unity.

*Upon receiving the results*,* I felt overwhelmed and consulted my elder siblings and uncles for guidance. (P4)*.

*My son and daughter-in-law provide strong support. My son insists he’ll manage medications and expenses*,* urging me not to worry. (P9)*.

Many caregivers reorient their lives around patients, intensifying familial bonds through sustained companionship.

*After her diagnosis, I ceased farming to focus entirely on her care. (P2).*

*I essentially video call them daily to check on their recovery and eating habits. We nearly return every week*,* fostering sense of heightened connection. (P13)*.

This enhanced family unity served as a vital source of strength and a key resource in managing the caregiving burden.

### Affirming patient value and dignity

Caregivers preserve patient dignity by respecting treatment preferences and acknowledging life achievements during illness. This validation reinforces patients’ self-worth amid health challenges. Maintaining the patient’s dignity and sense of purpose was not only crucial for the patient but also provided meaning and reduced distress for the caregiver.

*My father-in-low fully understands his condition and chose treatment at a renowned hospital. (P1).*

*He wants to grow vegetables like yams or peanuts in my brother’s yard. However*,* we hesitate to allow it*,* concerned it might affect his health. Still*,* sharing the harvest with us and the children brings him great joy and satisfaction. (P13)*.

*He maintains normalcy through chess outings, which we encourage. (P8).*

### Seeking professional guidance and support

Faced with the complexity of diseases and the necessity for emotional support for patients, caregivers often find themselves at a loss and urgently seek professional guidance from medical staff.

*My advice feels ineffective; I rely on clinicians to monitor her condition closely. (P2)*.

*If there is any discomfort*,* it is important to communicate with the physician as the doctors and nurses possess extensive experience. (P5)*.

*I hope the doctor can explain the treatment plan clearly and understandably*,* so that we can collaborate effectively in the future. (P12)*.

## Discussion

This study paints a vivid portrait of the “sky on the shoulders” of Chinese family caregivers for patients with advanced lung cancer, revealing a burden that is both multifaceted and profoundly shaped by cultural context. Guided by the Stress and Coping Framework, our analysis demonstrates that this burden extends beyond practical tasks to encompass profound informational deficits, compounded emotional and financial strain, culturally-shaped behavioral adaptations, and a critical reliance on support systems. These interconnected needs underscore significant gaps in the current provision of supportive and palliative care in China, where families remain the unchallenged cornerstone of long-term care yet receive insufficient systematic support.

### Profound informational needs as a foundational stressor

The profound need for information as caregivers grapple with illness-related uncertainty and fear is a universal theme in palliative care literature [15]. However, our findings further reveal a distinct pattern of passive reliance on physicians among Chinese caregivers. This reliance underscores how knowledge gaps and unmet information needs constitute a core stressor, contributing to misconceptions (particularly about chemotherapy), exacerbating emotional distress, and hindering adaptive caregiving. We found that even experienced caregivers reported persistent anxiety in the face of disease and treatment uncertainty, which consistently motivated them to seek more detailed information. These findings align with Zhang’s report [16]. A critical and novel insight from our study is the paradoxical decision-making observed: nearly one-third of caregivers consented to chemotherapy despite harboring significant fears and misconceptions about its effects. This finding highlights a crucial juncture where limited health literacy intersects with high-stakes medical decisions—a scenario not extensively reported in prior literature—and potentially compromises the process of truly informed consent, a cornerstone of patient-centered palliative care.

Prior research establishes that constrained disease knowledge can precipitate distress and hinder caregiving confidence [17, 18]. Our findings not only align with this but extend it by contextualizing the issue within the Chinese setting, revealing how factors such as lower educational attainment and digital illiteracy foster a distinct pattern of passive reliance on physicians’ recommendations [19]. In light of this, we emphasize that such passive reliance represents a missed opportunity for the shared decision-making central to palliative care. Therefore, we propose that palliative care teams should systematically assess caregivers’ health literacy and provide tailored information through accessible formats—such as visual aids or digital modules—to empower caregivers as informed and engaged partners, thereby alleviating anxiety and fostering more adaptive coping [20].

### The compounded emotional and financial burden, shaped by culture

Consistent with the Stress and Coping Framework, emotions emerged as direct outcomes of appraisal that reciprocally influenced subsequent coping. Our findings reveal that caregivers experience complex emotional trajectories—from initial shock and sadness to persistent anxiety, helplessness, and gradual acceptance—aligning with the findings of Ding [21] and Leow & Chan [22]. However, our study critically elucidates how, within the Chinese context, these universal challenges are profoundly intensified by cultural norms and socioeconomic pressures. Confucian values, positioning the family as the primary care locus, shaped emotional expression, leading to the suppression of personal distress to maintain harmony—a finding that nuances the general understanding of caregiver emotional labor. This cultural script also created poignant practical dilemmas, most notably around truth-telling. Families grappled with disclosure decisions burdened by fears of causing patient distress and concerns about social stigma [23], adding a significant layer of psychological strain.

Furthermore, this emotional burden was inextricably compounded by severe financial toxicity. Financial toxicity is increasingly recognized as a global and urgent determinant of family caregiver distress [24], with studies indicating that 17.6%–73.3% of cancer caregivers worldwide endure this burden [25]. Our findings confirm the high prevalence of financial toxicity in this setting, with over half of participants reporting severe strain and work-caregiving conflicts, which is consistent with the findings of Zhang, Zhou, et al. [26]. More importantly, they elucidate how its impact is uniquely magnified by the culturally-mandated, limitless sense of responsibility (filial piety) coupled with a less comprehensive social safety net. Culturally mandated caregiving roles often lead to employment disruption and loss of income, which, when combined with direct medical and ancillary costs, creates a debilitating financial burden [27]. Vulnerability is highest among those with limited education, sparse social support, or inadequate insurance [28, 29]. This intersection of emotional turmoil and financial precarity creates a synergistic stressor: financial worries exacerbate anxiety and depression, while emotional exhaustion can impair a caregiver’s capacity to navigate complex financial systems or maintain employment. Therefore, supportive interventions must be integrated and culturally attuned. Clinical practice should adopt a dual-focused approach, routinely screening for both emotional distress and financial toxicity, and providing concurrent support—including psychological counseling, communication guidance for dilemmas like truth-telling, and practical financial navigation aid [30].

### Cultural drivers, behavioral adaptations, and the need for skill-building

Our findings illustrate that caregiving behaviors are dynamically shaped by the interplay of cognition, emotion, and cultural context. Filial piety emerged as a core cultural driver, motivating dedicated care through constant companionship and fulfilling patient wishes, which resonates with the observations of Sun et al. [31]. However, our study also reveals a prominent contradiction within this cultural framework: caregivers simultaneously experience significant loneliness and a perceived lack of shared responsibility [32], indicating that the very cultural ethos that motivates care can also be a source of isolation.

This burden is markedly amplified during role adaptation and fraught communication. Although establishing effective communication is crucial, our data confirm that avoidance of sensitive topics and concerns about the patient’s psychological state often act as major barriers, aligning with previous research [33]. Notably, improving such communication plays a key role in fostering mutual respect and preventing conflict [34], highlighting the absence of skill-based guidance in current support systems.

A particularly significant finding relates to the caregivers’ own coping strategies. While they attempt to employ various methods (e.g., exercise, distraction), there is a widespread issue of “insufficient coping” and “coping misalignment.” More concerning is our identification of emotional suppression as a commonly adopted mechanism—one clearly linked to adverse health outcomes among caregivers [35]. This reliance on emotional suppression must be understood within its cultural context. Research indicates that the selection and, critically, the adaptiveness of emotion regulation strategies vary across cultures [36]. Specifically, while suppression may be more prevalent in East-Asian contexts for maintaining social harmony, its long-term link to poorer mental health highlights a problematic mismatch between cultural prescription and psychological well-being. Our findings provide a concrete, clinical illustration of this mismatch within Confucian familism and advanced cancer caregiving. Here, self-silencing is not merely a personal tactic but a culturally-prescribed duty, yet it remains maladaptive for the caregiver’s own health. This indicates a critical gap: caregivers are willing to cope but lack knowledge of and access to appropriate, effective, and sustainable strategies within their familial and cultural contexts. Palliative care interventions should therefore be designed to both build culturally-attuned caregiving skills and guide families toward a more adaptive model of “family harmony”—one that embraces open emotional expression and mutual support to sustain cohesion and protect caregiver mental health.

### The vital role of support systems as buffers

Our findings highlight strengthened family cohesion as a crucial buffer against caregiver burden, providing essential emotional and practical backing, consistent with research showing that familial harmony enhances resilience in adversity [37, 38]. Extending this understanding, the pivotal role we attribute to family cohesion strongly resonates with the international trend toward family-centered models in palliative care [39, 40]. More than merely affirming this approach in the Chinese context, our findings delineate the specific cultural mechanisms—such as collective problem-solving and reorienting life around the patient—through which family support becomes operational, offering a contextualized understanding of its protective function. Furthermore, caregivers play a key role in affirming patient dignity and meaning—a core function of palliative care that also enhances their own coping capacity [41]. Research corroborates that a sense of meaning and self-esteem in patients is linked to better psychological outcomes and quality of life [42, 43].

Both caregivers and patients expressed a strong desire for comprehensive, proactive professional support [44], a need that remains largely unmet in the current reactionary care model. To address the varying literacy levels and complex needs identified, healthcare systems must move towards integrated, multi-modal support. This includes strengthening tailored patient and family education, establishing in-hospital support groups, and critically, leveraging digital health. The “Internet Plus” model presents a significant opportunity to develop secure online support platforms offering just-in-time information, psychoeducational modules, relaxation training, and asynchronous peer support forums. Such a multifaceted system empowers caregivers to fully utilize available resources, ultimately improving their caregiving efficacy and ensuring continuity of care beyond the hospital walls.

### Implications for palliative care practice and research

To address the identified needs, clinical practice should: (1) routinely screen caregivers for distress, information needs, and financial burden; (2) provide culturally tailored support on communication, symptom management, and self-care; and (3) establish clear referral pathways to psychosocial and financial services. Concurrently, policy and research efforts should: (1) advocate for integrating and funding structured caregiver support, and (2) develop and test evidence-based, culturally tailored interventions, such as skill-building workshops and digital health solutions.

### Limitations

This study has several limitations. First, participants were recruited from a single tertiary hospital in Hebei Province, China, which may limit the transferability of our findings to caregivers in other regions with different healthcare resources and cultural norms. Second, although data saturation was achieved, the relatively small sample size (*n* = 14) may not capture the full spectrum of experiences among all family caregivers of advanced lung cancer patients. Third, the use of purposive sampling might have introduced selection bias, as caregivers who were more vocal or had stronger feelings might have been more likely to participate. Fourth, as a qualitative study based on a single interview, it provides a deep but cross-sectional snapshot of experiences. It cannot track how caregivers’ psychosocial needs and coping strategies evolve over the entire course of the illness. Future multi-center longitudinal studies across diverse socioeconomic and cultural regions in China are warranted to confirm our findings and track the evolution of needs over time.

## Conclusion

This study vividly illustrates the ‘sky on the shoulders’ of Chinese caregivers—a burden defined by profound needs and shaped by unique cultural forces. The identified multidimensional needs are not merely supportive but are fundamentally palliative care needs. Addressing them may require a significant shift in Chinese oncology care, one that integrates early, family-centered palliative care as a standard to systematically alleviate this burden, empower caregivers, and ultimately improve outcomes for both patients and the families who sustain them.

## Supplementary Information

Supplementary Material 1.

## Data Availability

The datasets generated and/or analyzed in this study are available from the corresponding author on request.The data are not publicly available due to privacy or ethical restrictions.
