# Supplementary material for: “The sky on our shoulders”: a qualitative study of family caregivers’ psychosocial experiences and unmet palliative care needs in advanced lung cancer
Source: BMC Palliat Care. 2026 Jan 20;25:43. doi: 10.1186/s12904-026-01991-8 (PMC12903627; doi:10.1186/s12904-026-01991-8)
Supplement: Supplementary file 1 — Supplementary Material 1. [file 12904_2026_1991_MOESM1_ESM.docx]

Table 1 Semi-structured interview questions

| No | interview questions |
| --- | --- |
| 1 | Since the patient's diagnosis,What information about the illness or caregiving have you found yourself searching for? What questions remain unanswered for you? |
| 2 | How has the role of being a caregiver affected your daily life, your work, and your relationships with other family members? |
| 3 | What negative emotions have you experienced during the caregiving process? How did you adjust? |
| 4 | What difficulties have you encountered while providing care? |
| 5 | What personal strategies or external resources have you found helpful in overcoming these challenges? |
| 6 | In the caregiving process, which areas do you anticipate healthcare professionals to provide support and assistance? |
